# Fluid Balance in the Critically Ill Child Section: “How Bad Is Fluid in Neonates?”

**DOI:** 10.3389/fped.2021.651458

**Published:** 2021-04-20

**Authors:** Austin Rutledge, Heidi J. Murphy, Matthew W. Harer, Jennifer G. Jetton

**Affiliations:** ^1^Department of Pediatrics, Medical University of South Carolina, Charleston, SC, United States; ^2^Department of Pediatrics (Neonatology), University of Wisconsin, Madison, WI, United States; ^3^Stead Family Department of Pediatrics (Nephrology), University of Iowa Health Care, Iowa City, IA, United States

**Keywords:** fluid overload, acute kidney injury, fluid balance, kidney replacement therapy, continuous renal replacement therapy, neonate

## Abstract

Fluid overload (FO) in neonates is understudied, and its management requires nuanced care and an understanding of the complexity of neonatal fluid dynamics. Recent studies suggest neonates are susceptible to developing FO, and neonatal fluid balance is impacted by multiple factors including functional renal immaturity in the newborn period, physiologic postnatal diuresis and weight loss, and pathologies that require fluid administration. FO also has a deleterious impact on other organ systems, particularly the lung, and appears to impact survival. However, assessing fluid balance in the postnatal period can be challenging, particularly in extremely low birth weight infants (ELBWs), given the confounding role of maternal serum creatinine (Scr), physiologic weight changes, insensible losses that can be difficult to quantify, and difficulty in obtaining accurate intake and output measurements given mixed diaper output. Although significant FO may be an indication for kidney replacement therapy (KRT) in older children and adults, KRT may not be technically feasible in the smallest infants and much remains to be learned about optimal KRT utilization in neonates. This article, though not a meta-analysis or systematic review, presents a comprehensive review of the current evidence describing the effects of FO on outcomes in neonates and highlights areas where additional research is needed.

## Introduction

In adults and pediatric patients, FO is associated with adverse outcomes including respiratory failure, cardiovascular events, prolonged hospitalization, and mortality (1–4). Recent studies suggest FO is similarly deleterious in neonates, but limited data are available. Neonates have unique physiologic renal adaptations, and neonatal fluid dynamics are complex. An understanding of the factors that impact fluid balance is required to prevent and/or treat neonatal FO as is a working knowledge of the available literature regarding associated morbidities and clinical outcomes.

### Postnatal Renal Adaptation and Fluid Dynamics

At birth the kidney is functionally immature; function slowly improves as renal blood flow and glomerular filtration increase during the neonatal period. In pre-term newborns, nephrogenesis is also not yet complete. Due to this functional renal immaturity, neonatal fluid dynamics are different from that of older patients, and quantifying fluid balance and detecting FO can be difficult. For example, total body water (TBW), which encompasses extracellular water (ECW) and intracellular water, is high in the fetus accounting for ~95% of body weight. Throughout gestation, the proportion of body weight represented by water decreases, but even newborns at term have TBW accounting for nearly 75% of their birth weight (BW) (5). After birth, isotonic contraction of the extracellular fluid compartment occurs with loss of ECW and accompanying weight loss. This physiologic diuresis is likely mediated *via* atrial natriuretic peptide. Regardless of gestational age (GA), newborns lose 10–15% of their BW in the first days of life and are then expected to regain their BW over the next 2 weeks (5, 6). Excessive fluid administration can confound this process and is associated with increased incidence of bronchopulmonary dysplasia (BPD), necrotizing enterocolitis (NEC), and patent ductus arteriosus (PDA) (7–10). In addition to measurable losses, insensible losses *via* the skin or respiratory tract can be considerable, especially in pre-mature neonates (6, 11). While fluid-based therapies are necessary for a variety of neonatal conditions, appropriate fluid regimens are highly debated. Fluid requirements are based on GA with differing hydration needs and nutritional goals for growth. With the expected volume contraction followed by restoration and varying insensible losses, establishing the ideal weight for use in fluid calculations can be challenging and requires ongoing careful evaluation utilizing weight changes, intake and output measurements, and serum and/or urine biochemistries.

### Fluid Balance Management Strategies

Fluid balance management strategies often include modifying environmental factors to minimize insensible losses rather than replacing estimated fluid losses given the risk of FO if estimates are incorrect. As described, excessive fluid administration can be harmful. Fluid restriction is another strategy to prevent complications associated with FO; however, presently available studies are inconclusive. A Cochrane review evaluating fluid restriction demonstrated a decreased risk of PDA and NEC in pre-term infants, but the five randomized controlled trials (RCTs) included are outdated and likely do not reflect current practices (12). In a more recent RCT, fluid restriction reduced the duration of respiratory support for severe transient tachypnea of the newborn (13). Conversely, Nicholson et al. found no difference in outcomes with post-operative fluid restriction in a cohort of neonates after cardiac surgery, a finding that suggests fluid restriction may not be helpful in all populations (14). Moreover, fluid restriction is not without consequences, risking adverse effects including dehydration, hypotension, and decreased end-organ perfusion. Optimal fluid therapy thus should allow for adequate postnatal diuresis with adjustment for increasing post-menstrual age as the kidneys mature and the degree of insensible losses diminish.

There is no consensus on how best to define FO (4), especially in neonates. FO is typically assessed by one of two methods: (1) weight-based methods, which quantify percent change in weight from baseline, or (2) cumulative fluid balance methods, which utilize daily fluid intake and output measurements from time of intensive care unit (ICU) admission (or other start point). Selewski et al. confirmed both methods correlate well in a cohort of pediatric patients receiving KRT (15). However, both approaches have disadvantages in neonates. First, the degree of postnatal diuresis and weight loss varies based on GA and confounds weight-based methods. Second, accurate recording of fluid intake and output is challenging. Van Asperen et al. found that fluid balance charted in the medical record correlated poorly with daily weight changes, and therefore providers may be unable to rely on recorded fluid balances as a sole measure in assessing FO in neonates (16). Third, diaper outputs are often recorded as “mixed” (consisting of both urine and stool), leading to uncertainty about how much of recorded volumes represent urine.

Further complicating fluid management is the co-occurrence of AKI with FO. Altered renal function secondary to AKI hinders diuresis after significant fluid accumulation, pre-disposing to the development of FO. Just as other biomarkers have been studied to better evaluate renal function (17), FO can be a marker of AKI (18). FO can also dilute Scr, hindering the ability to detect AKI. Once the existence of both disease processes is determined, it is difficult to differentiate and isolate the effects of AKI from FO; both can have profound, independent impacts on response to fluids and kidney function (19).

When FO is detected, it is unclear at what threshold treatment should be initiated in neonates. Specific FO thresholds ranging from 10 to 20% have been identified in older pediatric populations and adults as (1) requiring interventions and (2) associated with adverse outcomes (2, 36, 37). Unfortunately, similar thresholds have not yet been identified for neonates; depending on the clinical context, differing thresholds may exist.

### Fluid Overload Treatment Options

Treatment options for FO include diuretics, peritoneal dialysis (PD), and continuous KRT (CKRT). Diuretics are frequenty utilized in the ICU and are the mainstay of therapy for the prevention or treatment of pulmonary edema or congestive heart failure in infants with congenital heart defects (38). However, more research is needed to guide optimal diuretic dosing, timing, and type (loop vs. thiazide) in order to achieve desired outcomes without causing AKI or other organ injury. Belik et al. found diuretics improved pulmonary function in ventilated patients (39), though RCTs have failed to demonstrate improvement in outcomes in pre-term infants with respiratory distress syndrome, a precursor of BPD (40). Similarly, diuretics do not prevent the development or worsening of AKI in patients with oliguria, and the optimal use of diuretics to treat oliguria and FO in patients with or at risk for AKI is not yet clearly established (41).

Dialytic modalities including PD and CKRT are also options for fluid removal, but are not used as frequently for this indication in neonates, likely because of the lack of equipment designed specifically for small patients as well as lack of high quality data supporting optimal use. In the Assessment of Worldwide Acute Kidney Epidemiology in Neonates (AWAKEN) study, some form of dialysis was used in only 4.1% of those with AKI (42). Unlike diuretics, dialytic modalities offer the benefit of managing electrolyte imbalances while allowing for adequate nutrition provision that otherwise may be restricted in patients with oliguria and AKI. PD is often the preferred modality in neonates because of the avoidance of large fluid shifts and the need for large vascular catheters. A recent systematic review and meta-analysis by Flores et al. highlighted the challenges associated with using available data to guide clinical decision making around the use of PD (43). Their meta-analysis demonstrated an increased risk of mortality in patients who received PD post-operatively compared with those who were supported with diuretics, but a larger proportion of infants in this group came from centers that implemented PD following failed diuretic response and thus may represent a group at higher risk for poor outcomes. Outcomes by which efficacy of PD was assessed varied across studies, making cross-study comparisions difficult.

CKRT is another dialytic modality used to support neonates with FO and/or AKI. As mentioned previously, currently available machines have, until recently, been designed for adults and adapted for use in neonates. Studies assessing the outcomes of patients supported with CKRT consistently show higher mortality rates in patients <10 kg than in older and larger patients (44). The combination of technical challenges and published rates of high mortality likely contribute to hesitancy around the use of this therapy in neonates. However, newer devices with lower extracorporeal volumes are now becoming available. Menon et al. published multi-center data on the adapted use of the Aquadex FlexFlow system (CHS solutions Inc., Eden Prairie, MN) (45). In their study, FO was the indication for this therapy in 46% of their sample and FO with AKI in another 15%. Even with this smaller circuit, survival rates were lower in patients <10 kg than in the other patient groups, with 60% of patients <10 kg surviving to treatment discontinuation compared with 97–100% in older/larger patients (overall survival: 32% in patients <10 kg vs. 68–85% in older/larger patients). The Cardio Renal Pediatric Dialysis Emergency Machine (Carpediem^™^) is the only machine specifically designed for neonates and recently received FDA approval for treatment of AKI and FO in patients 2.5–10 kg. Published survival outcomes for neonates with AKI and FO are higher using this machine, with 97% of the sample surviving to treatment discontinuation, and 50% overall survival of this group (46). These newer devices have significant potential to expand our therapeutic options and improve our ability to manage FO (and AKI) in neonates.

## Subsections: Specific Populations at Risk for FO

Below is a brief review of key studies (Table 1) of FO in several specific neonatal populations: those with cardiac disease or requiring cardiac surgery, those with lung disease, and those requiring extracorporeal life support (ECLS) and CKRT Table 1.

**Table 1:** Impact of fluid intake, FO, and weight loss on outcomes in neonates.

**References**	**Study design**	**Population**	**Findings/observations**
**PDA and cardiac surgery**			
Stevenson et al. (47)	Case control study	62 infants with RDS BW <2,000 g	Infants who developed PDA received significantly increased daily fluid intake in the 2 days prior to diagnosis (*p* = 0.001).
Brown et al. (20)	Case control study	105 infants with RDS	Total fluid intake during the first 5 days of life was significantly higher in infants with PDA (*p* < 0.025).
Bell et al. (8)	RCT	170 infants, BW 751–2,000 g	• Increased risk of PDA in the high fluid intake group (i.e., >20 mL/kg/day above the upper limit of low fluid group) (*p* < 0.001). • No significant difference in BPD or mortality.
Stephens et al. (21)	Retrospective cohort study	204 infants <32 weeks GA, BW ≤1,250 g	Fluid intake on days 2–3 of life was an independent risk factor for PDA (OR 1.014, 95% CI 1.001–1.040).
Hazle et al. (22)	Prospective cohort study	49 infants <6 months who underwent congenital heart surgery with CPB	• Max FO by both weight-based (*p* = 0.03) and fluid balance (*p* = 0.02) methods was associated with composite poor outcome (need for CKRT, duration of MV, ICU length of stay, mortality). • Infants with poor outcomes were more likely to be neonates (*p* = 0.001).
Piggott et al. (23)	Retrospective cohort study	95 infants who underwent cardiac surgery	FO >15% was associated with increased mortality (*p* < 0.001), longer duration of hospitalization (*p* = 0.03), and longer duration of MV (*p* < 0.001).
Wilder et al. (24)	Retrospective cohort study	435 infants who underwent cardiac surgery with CPB	FO >16% on post-operative day 3 was an independent risk factor for composite poor outcome of need for CKRT, ECLS, or death (*p* < 0.01).
Mah et al. (25)	Retrospective cohort study	117 infants who underwent cardiac surgery with CPB	FO was independently associated with mortality (*p* = 0.032), length of hospitalization (*p* < 0.001), and length of cardiac ICU stay (*p* < 0.001).
**Lung function, development of BPD, and need for MV**			
Palta et al. (26)	Retrospective cohort study	220 infants from the Newborn Lung Project, BW <1,200 g	PDA (*p* = 0.003) and mean daily fluid intake over the first 4 days of life (*p* < 0.01) were associated with development of BPD.
Oh et al. (27)	Retrospective cohort study	1,382 infants from Neonatal Research Network RCT, BW 401–1 000 g	Higher daily fluid (mL/kg) intake (*p* < 0.001) and less weight loss from BW (*p* = 0.006) during the first 10 days of life was associated with increased risk of BPD or death.
Van Marter et al. (9)	Case control study	223 infants from a RCT of phenobarbital prophylaxis for intracranial hemorrhage	Infants with BPD received greater total daily fluids adjusted for BW (*p* < 0.05) and demonstrated an average net weight gain (*p* < 0.05) compared with net weight loss in control infants during the first 4 days of life.
Marshall et al. (10)	Case control study	865 infants, BW 500–1,500 g	Higher daily fluid (mL/kg) intake during the first 5 days of life was associated with the development of BPD (*p ≤ * 0.001).
Wadhawan et al. (28)	Retrospective cohort study	9,461 infants from the Neonatal Research Network database, BW <1,000 g	Early postnatal weight loss was associated with decreased risk of BPD or death (OR 0.51, 95% CI 0.42–0.62).
Matsushita et al. (29)	Retrospective cohort study	219 infants, BW <1,000 g	FO >15% was significantly associated with mortality (*p* = 0.002) and longer duration of MV (*p* = 0.002).
Selewski et al. (30)	Retrospective cohort study	645 infants >36 weeks GA from the AWAKEN study	Peak FB during the first postnatal week (*p* < 0.001) and FB on postnatal day 7 (*p* < 0.001) were independently associated with need for MV on postnatal day 7.
Selewski et al. (31)	Retrospective cohort study	1,007 infants <36 weeks GA from the AWAKEN study	Peak FB during the first postnatal week (*p* < 0.001) and FB on postnatal day 7 (*p* < 0.001) were independently associated with need for MV on postnatal day 7.
**ECLS and CKRT**			
Selewski et al. (32)	Retrospective cohort study	756 pediatric patients (60% neonates) who required ECLS for >24 h	• FO >20% at initiation of ECLS (*p* < 0.001) and peak FO >30% on ECLS (*p* < 0.001) both predicted mortality. • The neonatal population had a higher peak FO on ECLS (*p* < 0.001).
Selewski et al. (33)	Retrospective cohort study	53 pediatric patients (62% neonates) who underwent concurrent CKRT and ECLS	• FO at CKRT initiation predicted mortality (OR 1.04, 95% CI 1.01–1.08, *p* = 0.018). • No improvement in survival with FO correction to <10% (OR 1.22, 95% CI 0.13–11.1, *p* = 0.860). • Neonates had significantly lower rate of survival to ICU discharge (15.1 vs. 65%, *p* < 0.001).
Lee and Cho (34)	Retrospective cohort study	34 infants with AKI who required CKRT >24 h	FO ≥ 30% at initiation of CKRT was associated with worse survival (*p* = 0.009).
Gorga et al. (35)	Retrospective cohort study	357 pediatric patients (52% neonates) who underwent concurrent CKRT and ECLS	FO at CKRT initiation was significantly lower in survivors (13.5 vs. 25.9%, *p* = 0.004) and associated with longer duration of ECLS (*p* = 0.01).

*PDA, patent ductus arteriosus; RDS, respiratory distress syndrome; BW, birth weight; g, grams; RCT, randomized controlled trial; mL/kg/day, milliliters/kilogram/day; BPD, bronchopulmonary dysplasia; GA, gestational age; OR, odds ratio; 95% CI, 95% confidence interval; CPB, cardiopulmonary bypass; FO, fluid overload; CKRT, continuous kidney replacement therapy; MV, mechanical ventilation; ICU, intensive care unit; ECLS, extracorporeal life support; mL/kg, milliliters/kilogram; AWAKEN, Assessment of Worldwide Acute Kidney Epidemiology in Neonates; FB, fluid balance; h, hours; AKI, acute kidney injury*.

### PDA and Cardiac Surgery

Persistence of the ductus arteriosus (i.e., PDA), the most common cardiac problem among pre-term infants, is associated with excessive fluid intake (8, 20, 21, 47). Fluid intakes on days 2 and 3 of life are independently associated with increased risk of PDA indicating that early fluid administration can be problematic even before FO develops (21). In this same study, the odds of PDA increased 22% for every 10 mL/kg of fluid received on day 3, and those who received total fluid intakes >170 mL/kg/day were 4.5 times more likely to have a PDA.

Cardiac surgery is a risk factor for FO as well as AKI. Both complications often occur with cardiopulmonary bypass (CPB) support and are associated with significant morbidity and mortality (22–25). Peri-operative fluid management is complicated by the need for blood products and diuretics, impaired renal function, and low cardiac output necessitating volume resuscitation balanced with inotropic/vasopressor support. In a retrospective cohort of neonates undergoing CPB, FO was an independent risk factor for the composite outcome of death and need for CKRT or ECLS (24). Notably, those with poor outcomes were more likely to be <3 days old at the time of the operation signifying acuity of illness but also possibly reflecting inadequate diuresis pre-operatively. The authors determined that >16% FO on post-operative day (POD) 3 held the highest predictive value for poor outcomes, suggesting POD 3 and a FO >16% could represent important therapeutic thresholds. FO can also impact recovery by prolonging the duration of mechanical ventilation, time to sternal closure, and overall length of stay in neonates after cardiac surgery (23, 24). Mah et al. have also demonstrated the independent association of FO on length of stay as well as mortality (25).

### Lung Function, Development of BPD, and Need for Mechanical Ventilation

Most research evaluating the relationship between fluid balance and the lung in neonates has focused on ELBW infants and BPD-related outcomes. Multiple factors are implicated in the pathogenesis of BPD including barotrauma, oxygen toxicity, and PDA; positive fluid balance and subsequent pulmonary edema are proposed to contribute as well (20, 26). Researchers hypothesize excessive fluid administration leads to increased pulmonary blood flow (especially if PDA is present), and this excess fluid subsequently shifts from the vessels into the pulmonary interstitium. Resultant pulmonary edema negatively affects lung compliance, requiring increased respiratory support and risking potential lung injury (27, 48).

Multiple studies have found increased risk of BPD in infants who received higher total fluid intakes and less postnatal weight loss through the first 10 days of life (9, 10, 27, 28). More recent investigations explored the link between neonatal fluid balance and mechanical ventilation and found FO in the first 72 h of life was associated with higher ventilator settings and longer duration of mechanical ventilation (29). In a secondary analysis of the AWAKEN cohort (42), Selewski et al. demonstrated multiple measurements of positive fluid balance as risk factors for the need for mechanical ventilation at the end of the first week of life (30, 31); every 1% increase in peak fluid balance led to a 12–14% increased risk of requiring mechanical ventilation on postnatal day 7, suggesting even incremental fluid changes can adversely affect lung function.

### ECLS and CKRT

Neonates requiring ECLS are the most critically ill patients in the neonatal ICU. Severe respiratory pathology is a common neonatal indication for ECLS, and FO with pulmonary edema can be detrimental to the recovery of lung function (49). FO is common in pediatric patients requiring ECLS with the majority developing >30% FO in one multi-center observational study (32). Although this study included all pediatric patients, the median patient age was 10 days. Positive fluid balance alone during ECLS was an independent risk factor for mortality, and neonates had significantly higher peak FO (35.3 vs. 26.3%; *p* < 0.001) than pediatric patients.

CKRT is often used in patients receiving ECLS to prevent or treat FO. In another retrospective study examining pediatric ECLS patients comprised of 62% neonates, Selewski et al. demonstrated increased risk of mortality with a higher degree of FO at CKRT initiation (33). Although CKRT may be able to improve fluid balance, the authors also showed that once FO was established, fluid removal had no impact on survival (33). This associated risk of mortality was later reiterated in an exclusively neonatal observational study using a FO threshold of 30% (34). Gorga et al. conducted a multi-center cohort study in which neonates represented 52% of the sample and also found an independent association between the degree of FO at CKRT initiation and mortality with graded increases in both ECLS and hospital mortality for every 10% increase in FO (35). However, the median change in FO from CKRT initiation to discontinuation did not seem to impact either ECLS or hospital mortality. The mortality risk with positive fluid balance on ECLS and the lack of survival benefit with fluid removal suggests earlier recognition and intervention with CKRT may be indicated prior to the development of FO. However, the threshold at which this should occur remains unknown.

## Discussion and Conclusions

Neonatal fluid dynamics are complex, evolve throughout the neonatal period, and present clinical challenges. Neonatal fluid balance is impacted by a variety of factors, and FO can be difficult to quantify accurately and reliably. Moreover, FO occurs frequently in high-risk neonates including those with cardiac disease, lung disease, and those requiring ECLS, and is associated with worse outcomes. In addition, neonates with AKI and FO requiring dialytic support consistently have higher mortality rates across studies than do older and larger patients. Whether it is the FO itself, the combination of AKI and FO, or an overall increased severity of illness for which FO may simply be a marker is not yet known.

It is also unclear whether FO is a modifiable risk factor. That is, can we improve outcomes by preventing fluid accumulation? If so, what are the thresholds at which to intervene? As we recognize the harmful effects of FO in neonates, additional research is warranted to evaluate this relationship. Research efforts will be enhanced by the standardization of definitions of FO, methods by which to quantify fluid balance (recognizing that these may vary by GA and day of life), and ideal outcomes by which to assess interventions (e.g., time to extubation, ICU and hospital length of stay, AKI occurrence and duration, and mortality). Furthermore, multi-center RCTs are needed to guide clinical management by identifying therapeutic thresholds at which to intervene, and the indications for and appropriate use of FO treatment strategies (both diuretics and dialytic modalities). Finally, the impact of these treatment strategies on long-term outcomes will need to be explored. Greater awareness of this important clinical issue at the bedside, enhanced research focus, and the availability of dialysis equipment designed specifically for neonates have the potential to expand treatment options and improve outcomes for these vulnerable patients.

## Author Contributions

AR is responsible for the conception, design, drafting, and completion of this review. HM, MH, and JJ have assisted with analysis and interpretation of his work and critically revised this manuscript. All authors are fully accountable for ensuring the integrity and accuracy of this work and have approved this final version.

## Conflict of Interest

The authors declare that the research was conducted in the absence of any commercial or financial relationships that could be construed as a potential conflict of interest.
